# On the ethics of healthy ageing: setting impermissible trade-offs relating to the health and well-being of older adults on the path to universal health coverage

**DOI:** 10.1186/s12939-019-0997-z

**Published:** 2019-09-05

**Authors:** Kebadu Mekonnen Gebremariam, Ritu Sadana

**Affiliations:** 1 Department of Philosophy, Addis Ababa University, Algeria St, P.O.Box 1176, Addis Ababa, Ethiopia; 2 Ageing and Life Course, World Health Organization, 20 Avenue Appia, 1211 Geneva 27, Switzerland

**Keywords:** Healthy ageing, Impermissible trade-offs, Dignity, Autonomy, Living-well

## Abstract

This article aims to clarify the moral underpinning of the policy framework of *Healthy Ageing*. It is a policy adopted by the World Health Organization designed to operate in alignment with the United Nations (UN) framework of the Sustainable Development Goals (SDGs) and the urgency given for the achievement of Universal Health Coverage (UHC). It particularly reflects on what, if anything, justifies protecting the most basic rights to health and well-being of older adults from possible policy trade-offs on the path to UHC.

It argues that the dignity of older adults―under which are nested more specific ideas of self-respect, respect for autonomy, as well as the ethical priority for living well―underpins a categorical moral injunction against imposing the familiar utilitarian calculus as the default criterion for policy trade-offs across age groups. Respect for the dignity of older persons marks the moral threshold that every society ought to uphold even under conditions of relative resource scarcity.

The moral constraint on permissible policy trade-offs relating to the health of older adults must reflect an understanding of older persons as active agents in the social structure of (their) well-being, not merely as passive vessels through which a good healthy life may or may not occur. We argue that there are three main domains where trade-offs are unacceptable from the moral point of view: it is impermissible (1) to prioritise key service(s) across different (vulnerable) age groups on the basis of actual or future contribution to society, (2) to prioritise across different age groups when co-prioritisation is warranted by the ethical theory, and (3), to always prioritise (by default) services that improve well-being over those that foster respect for dignity and autonomy.

## Introduction

### Context and focus

#### Demographic landscape

Research has indicated that as of 2018 the number of older persons aged 60 or over reached 1 billion for the first time [1] and, projections showed that, by the year 2030 there will be more older people than the number of children under 9. While in 2015 about 13% of the global population was 60 years and above, which is expected to almost double by 2050 and to more than triple by 2100, rising from 962 million in 2017 to 2.1 billion in 2050 and 3.1 billion older adults in 2100 [2]. That means by the year 2050 there will be 106 older persons for every 100 children in the world. In China, for example, the proportion of older persons is expected to rise to 33.9% of the total population in 2050 (SAGE China Wave 1), whereas in India the proportion is expected to reach 19% by 2050 (SAGE India report). Regional differences are also projected to widen, where for instance European population aged 60-plus will hit 34% by 2050 while, in contrast, the proportion in sub-Saharan Africa is expected to be much lower [3].

In developing countries, increased life expectancy is due largely to actions against the major causes of mortality at younger ages, the success of which is made sustainable by development programs that helped raise hundreds of millions of people out of abject poverty. The convergence of significantly reduced infant and maternal mortality with an improved overall wellbeing results in the fact that most people can now expect to live longer. In high income countries where life expectancy has been on the ascending since the industrial revolution, continuing increases in longevity are now mainly attributed to the improvement and maintenance of well-being at older ages.

This demographic transition to older population age structures will certainly catalyse profound social and economic changes and challenges, with direct implication for health and sustainable development exacerbating the already complex problems regarding the establishment of just and sustainable institutions. On average the global life expectancy at age 60 is estimated to be 20 additional years [4]. However, global averages are deceptive as they mask absolute differences in health status across and within countries with respect to life expectancy as well as risk to disease and disabilities at older age. Research indicated that between countries there is a range of 38 years for life expectancy at birth, 37 years for healthy life expectancy at birth, and 13 years for life expectancy at age 60 and above [5]. In a similar vein, care needs are far higher for people older than 65 years living in sub-Saharan Africa than people of similar ages in more developed countries. According to WHO estimates, in Ghana more than 50% of people between the age of 65 and 75 years require assistance with daily activities, while the percentage jumps to 65% for those 75 years and older. In South Africa, more than 35 and 45% respectively require assistance; whereas in Switzerland, of those at similar ages, the percentage is less than 5 and 20%, respectively [6]. As these examples demonstrate, at older age the people with the greatest health needs tend to also be those with the least access to institutional, social and financial resources that might help to meet them [7]. Most of the available care for older persons in sub-Saharan Africa is provided by families where female relatives constitute the overwhelming majority of care givers [8]. The upshot is that, given existing health inequities partly driven by socio-economic disadvantages accumulated over the life course, urgent action is needed at all critical stages in life, without at the same time neglecting the legitimate claims and most pressing needs of older adults [9].

##### **Commitments**

As part of the effort at operationalising the UN Sustainable Development Goals (SDGs) that are of particular relevance to health, such as “Goal 3: Ensure healthy lives and promote wellbeing for all at all ages”, all UN Member States unanimously pledged to commit to the achievement of universal health coverage (UHC) by 2030. UHC is consequently defined as a system of ensuring that all people can use the promotive, preventive, curative, rehabilitative and palliative health services they need, of sufficient quality to be effective, while also ensuring that the use of these services does not expose individuals to financial hardship [10]. Achieving UHC is one of the key policy objectives set by the global community aimed at ameliorating the critical gap that figures the commitment to ensure the highest achievable health *at all ages* is measured against the reality of extreme inequalities in access to health within and across societies. According to the World Health Organization’s factsheet on UHC, the figures for 2016 indicate that at least 400 million people globally lack access to one or more essential health services. In addition, every year an estimated 100 million people are pushed into poverty and 150 million people suffer financial catastrophe due to out-of-pocket expenditure on health services, wherein out-of-pocket payments constitute on average 32% of each country’s health expenditure [11]. Households with older persons over the age of 50 are often hit by financial hardships much harder than households with younger age groups. That is because ageing correlated multiple morbidities and the corresponding need for health care is unmatched by insufficient resources owing to the gradual decline in household income. Despite that and contrary to conventional wisdom, scientific data indicated that the ageing of people could be affordable and could be of great benefit for both developing and developed countries due to the social capital of older adults as well as accrued wealth across the longer life-course (hence the talk of a third demographic dividend) [12].

##### **Health policies and strategies**

The stated global commitment to ensure UHC subsequently requires that each country establishes a fair and equitable path to UHC through a national strategy and action plan that operates in alignment with the global commitments, while at the same time being specific enough to respond to the country’s unique situation. In response to challenges specific to improving the health and well-being of older adults, the World Health Organization sought to provide technical support by producing a number of global strategic frameworks, reports and policy guidelines.

Some of those documents specifically respond to the most pressing need to render existing services sufficiently inclusive of older adults, which does not just mean providing them with greater access to services that are designed for younger adults [13]. Whereas a just distribution of resources can be effected within nations and efficiency improved, for most countries the ratio of resources to need will nevertheless remain to have significant disparity. In balancing such disparity, critical choices are to be made concerning which services to prioritise and who to include first. That requires a principled guideline on setting priorities and on distinguishing the set of services that can possibly be traded-off from those services deemed to be most basic that their provision is morally required under all conceivable circumstances.

Since this article is principally concerned with demarcating the moral boundaries of permissible trade-offs involving the health and wellbeing of older persons, three principal WHO documents immediately fall within its purview. These are, the final *Report of the WHO Consultative Group on Equity and Universal Health Coverage (2014),* the 2015 *World Report on Ageing and Health*, and the *Global Strategy and action plan on ageing and health (2016)*. This is not to say that regional policy frameworks or national legislations relevant to older people have little significance for strengthening the rights to health and wellbeing of older persons. However, most regional or national policies and legislations overlook significant aspects of the need to put in place a principled path to priority setting and policy trade-offs. Although national constitutions and regional protocols provided an important framework for anchoring the rights of older persons, only a handful made explicit reference to older persons’ protective status–for instance, the Constitution of Philippines designates older people as a priority group, while underscoring the need for employing a holistic approach to their health (Government of the Philippines, 1987).

Ethical guidance is required precisely because no overarching framework for priority setting and trade-offs is available that is specific to the right to health of older persons. Whereas the WHO framework on trade-offs is grounded on prioritising services not age groups, and that a reference to prioritising the ‘worse-off’ often blurs the distinction between economic inequalities and inequalities in health status on the one hand, and between absolute and relative worseness on the other.

##### **Ethical guidance**

The ethical guidance that this article advances shall draw from important conceptual distinctions in moral philosophy; more substantive debates regarding health inequalities and inequities and what it means to optimise the use of limited resources; and from moral and legal analyses relating to the normative implications of defining health as a right. We shall not carry out a comprehensive theoretical repackaging of all WHO documents relating to ageing and health, only those whose underlying ethos is relevant to priority setting. To that effect, we recognise that a comprehensive conceptual groundwork is needed, a task that admittedly lies outside the scope of this article. We do, however, believe that the arguments advanced here should be central to such undertaking and the analytical approach used here sets the tone for the future task of developing a comprehensive normative framework underpinning the WHO framework for ageing and health.

The proposed ethical guidance begins by expounding the overarching conceptual scheme within which the WHO’s operative term―*Healthy Ageing* is to be found, then identifies the specific area where the WHO’s approach to ageing and health needs some basic ethical guidance. It highlights the urgency not to lose sight of important ethical or moral concerns attached to how older persons ought to be treated, including which interests of theirs to be taken seriously by their fellows, either in their personal standing as free and equal members of the moral community or in their shared fellowship and the solidarity expressed through the apparatus of the state.

There is a perceptible gap between the WHO’s conceptual model for *Healthy Ageing* and the aforementioned WHO documents that confer policy guideline. The more substantive part will then attempt at bridging that gap; it does that first by making explicit the unspoken assumptions of the theoretical model for *Healthy Ageing*. Then it sets up a moral theory against which the WHO framework and its unspoken assumptions can be weighed. Having done that, the established moral theory will finally mount a defence of specific policy trade-offs that it identifies as impermissible from the moral point of view. Impermissibility is a condition of complete moral prohibition that admits no degrees. If something is declared impermissible, it implies that whatever is prohibited must not under any (non-catastrophic) circumstances be violated.

## Establishing the conceptual groundwork for the framework of healthy ageing

### Three cardinal questions

Towards the end of his first ground-breaking work―the *Critique of Pure Reason*, the German philosopher Immanuel Kant stated that the substantive kernel of any philosophical endeavour, whether speculative or practical, boils down to resolving either of the following three fundamental questions: “what can I know?”, “what ought I to do?”, and “what may I hope for?”. The first question is principally epistemic, in the sense that it examines the nature, scope and justification of knowledge under which can operate any particular field of inquiry. Answers to the question “what ought I to do?” settles practical considerations that pertain to the nature and the proper motive for a course of action that one is either permitted, obligated, or for that matter forbidden, to do.

That is precisely where thin concepts such as ‘right and wrong’, ‘good and bad’, ‘permissible and impermissible’, and ‘respect and violation’, or substantively thick concepts such as fairness, justice, kindness, generosity, selfishness, and impartiality find their proper reflective platform. And thirdly, Kant reasoned that “what ought I to do?” engenders a deeper question of meaning and significance of acting as one ought―a philosophical investigation that is both speculative and practical all at once.

Most philosophical problems are composite in nature for they include a mix of each of the three questions, their difference is mainly in emphasis. Similarly, we must recognise that the aforementioned WHO documents on *Healthy Ageing* cut across the three Kantian lines of philosophical questioning. However, for the sake of simplicity, we can describe what each document principally seeks to investigate in terms of one of the three philosophical questions that each document predominantly seeks to answer. The *World report on ageing and health* systematically captures *what we can and do know* about ‘ageing and health’, and in so doing further articulates the challenges presented by the rapidity with which the world’s population is ageing. Drawing from a body of knowledge emanating from multidisciplinary research on ageing and health, the report identifies key areas of immediate concern “and builds a strategic framework for taking public-health action, with a menu of practical next steps that can be adopted for use in countries at all levels of economic development.” [14]

The *Global Strategy and action plan on ageing and health*, on the other hand, makes explicit the principles that underpin an adequate national strategy and plan of action necessary for fostering *Healthy Ageing*. To fulfil its vision for realising a world in which everyone can live a long and healthy life (‘what may we hope for’), and focusing on five strategic objectives, the *Strategy* seeks to implement 5 years plan of action (2016–2020) for ensuring a Decade of Healthy Ageing from 2020 to 2030 at which point “functional ability is fostered across the life course and where older people experience equal rights and opportunities and can live lives free from age-based discrimination.” [15] The underlying principles are: human rights, equity, equality and non-discrimination on the basis of age, gender equality, and inter-generational solidarity. Whereas the key areas for national actions that the *Strategy* sets out to accomplish include establishing national frameworks towards *Healthy Ageing,* strengthening national capacities to formulate evidence-based policy, and combating ageism.

In a nutshell, what may we hope to achieve is the highest achievable health for all through the scheme of Universal Health Coverage and optimize healthy ageing. What ought we to do? A generic reply could be, ensuring a fair and equitable path to UHC that is inclusive of older persons. And that is precisely what the third WHO document ― the final *Report of the WHO Consultative Group on Equity and Universal Health Coverage* ― seeks to establish. It recognises that critical choices are to be made as to which services to prioritise, whom to include first, and how to shift from out-of-pocket payments towards a system of prepayment that does not render getting needed and effective services conditional upon the person’s ability to pay. Consequently, the report identifies three areas of strategic action: (1) categorising services into priority classes―guided by principles of cost-effectiveness, priority to the worse off and financial risk protection; (2) expanding coverage for high-priority services to everyone while devising fair and equitable ways of eliminating out-of-pocket payments; and (3) ensuring that disadvantaged groups are not left behind.

The path to UHC is an arduous journey that involves continuous improvement, since each country experiences some form of resource or structural constraint, requiring prioritisation and trade-offs at every step of the way. In moving towards a progressive realisation, some trade-offs are therefore unavoidable. With that in mind, the *Report on Equity and UHC* identifies five particular scenarios in which trade-offs are generally unacceptable. The first three unacceptable trade-offs correlate to the first strategic action and its attendant principles, whereas the other two correspond to each of the remaining two strategic actions.

In contrast to the first two WHO documents, the final *Report on Equity and UHC* is construed in general terms and thus makes no specific reference to older adults. But substantively the latter hits the right notes for it concerns itself with the topic of fairness and trade-offs on the path to UHC. In addition, the *Report* identified five generic impermissible trade-offs that national policies should dispense with on the path to UHC. Since our specific concern is identifying which of the legitimate concerns for the health and well-being of older adults are impermissible for trade-offs, that poses some methodological problems. In settling our specific concern for older adults, two possible analytic approaches can be taken.

One approach may be to identify unacceptable trade-offs attendant upon the health of older persons on the grounds of fairness (and the overlapping concern for equity), and then fine-tune the list so that it aligns with the ethical reasoning that underpins the five specific trade-offs that the *Report on Equity and UHC* declared unacceptable. Even though *the report on Equity and UHC* addresses health policy issues affecting societies across the board, one may argue that we can identify those issues and concerns that uniquely affect older persons on similar, equity-based, grounds. Such analytic route runs the risk of being procrustean, in the sense that it might compel us to stretch the normative scope of fairness under which are to be nested all fundamental moral concerns relating to the health and well-being of older persons. And, on the flip side, this approach appears to neglect some impermissible trade-offs that are grounded on moral principles that do not in the first instance reflect equity or fairness.

Or we could proceed in the other direction: begin by independently formulating valid ethical and moral standards that warrant stringent normative constraints against trade-offs on the health and well-being of older persons, then look to see if we find compelling the concrete convictions about unacceptable trade-offs itemised in *the report on equity and UHC*. This potentially permits the deployment of basic ethical and moral judgments, that are nested on principles other than the requirement of fairness, in defence of the claim that some of the specific rights to health and well-being of older persons are impermissible for trade-offs. This appears to be a promising approach for discovering some compelling moral reasons for protecting the claims of older persons, claims that do not figure in existing policy documents; but the judgements thus established need to be oriented towards policy making.

The required moral framework should therefore pursue an integrated approach that allows for examining the specific concern for fairness within the broader moral reasoning about what we owe to older persons.

## A PATH to UHC inclusive of older adults: a theoretical overture on trade-offs and impermissibility

Moral norms and principles that underpin UHC as a critical policy framework that every country ought to adopt may differ from ethical considerations relevant for regulating UHC’s progressive realisation. Progressive achievement of Universal Health Coverage implies prioritisation of services and deciding who to include first, further requiring a principled approach to permissible trade-offs when two or more priority items compete for primacy given actual structural or resource constraints. We ought to first identify the key concepts pertinent to such demarcation.

The concern for setting the moral boundaries to permissible trade-offs relating to the health and well-being of older persons can be attached to the specific domain of morality within which impermissibility is to be found. Ethical and moral standards are characterised by a set of considerations or normative conditions that establish what is morally permissible, required, or is impermissible to do. What is morally permitted for a person to do may or may not be morally required. But all things that one is morally required to do must also be permitted, otherwise the entire moral enterprise would be self-defeating. Impermissibility is a special variation of moral requirement that invokes a unique set of principles. Moral imperatives differ in their normative force, and impermissibility is a normative condition that warrants the stringent moral force. It is often invoked in the discourse on basic human rights, specifically attached to respect for life and liberty of persons and to the notion of respect for human dignity. Moral impermissibility reflects the unique purchase that basic moral imperatives have in guiding practical life, precisely, as *normative side-constraints* on the rational pursuit of either individual or otherwise collective ends.

Clearly, the public-health framework of *Healthy Ageing* needs to make certain generalisations, whereas the moral side-constraint we seek to prescribe makes sure that policy choices do not violate the rights of individuals. How can we then decide about the right path to UHC that is inclusive of older adults, which at the same time does not infringe on their fundamental rights? Again, providing the full answer to this question is beyond the scope of this article, it only seeks to show with reasonable certainty the wrong path that the public-health framework should avoid. We can certainly know what is plainly unjust or morally wrongful without, at the same time, committing ourselves to declaring with finality what, for a particular country, the perfect path to UHC would be like.

In what follows we clarify relevant conceptual distinctions crucial for developing the underlying ethical framework which allows us to identify impermissible trade-offs on the rights to health and wellbeing of older persons.

### Two distinctions in ethics and morality

#### How ought we to live

The terms ethics and morality are often used interchangeably both in the academic philosophical literature as well as in practical policy instruments. In their strict senses, however, the “ethical” and “moral” connote distinct meanings admitting subtle but meaningful normative differences than meets the eye. Morality concerns with how we ought to treat each other and our duties in this regard, whereas ethical standards prescribe how ought we to live ourselves if we are to ‘live well’ and have a ‘good life’. It must, however, be noted that distinction does not entail separation. Ethics and morality share the exact same normative sphere in the same way the two heads of conjoined twins (of ‘dicephalic parapagus’ sort with shared vital organs) may share the same body frame below the neck. Although dicephalic twins are considered as distinct persons in their own right, each twin cannot claim absolute sovereignty over the lower body and for that reason cannot survive without the other. So in the same way, ethics and morality maybe the two separate heads of the same normative value system.

The received view asserts that ethical life partly requires observing moral principles such that how “I ought to live certainly encompasses my concern with how I ought to treat others.” But scholars often disagree over the extent to which our moral responsibility towards others informs our ethical responsibilities to ourselves. In a nutshell, two implications obtain from the conceptual nexus between the ethical and the moral:

One suggests that how I should value my life is not ethically optional in the same way my duties to others are not; and secondly, my ethical responsibilities towards myself confers an overriding reason for me to act such that I am not required to neglect how my life goes in order to fulfil my moral obligations to/regarding others [16].

According to Ronald Dworkin, “my concern with how ought I to live must in some sense have an overriding reason for me to act, such that the ultimate value to my personal life is neither exclusively nor primarily a function of how I treat others.” [17] And secondly, morality connects with human aspiration quite positively given that we conceive of our ethical responsibility to live well as generating the scope of morality’s constraining power in practical life.

##### **Living well and the good life**

The above sketched relation between ethical and moral value judgments can be systematically explained in terms of a distinction within ethics that is also familiar in morals, namely the distinction between the right and the good―between living well and having a good life, between duty and consequence respectively. The main take away is that, the value to one’s life is measured not only by the amount of a good life that it produces either to oneself or to others, but primarily in how rightly it was lived irrespective of its product value. Living well consists in the performance value, while the good life is characterised by the end product that life bestows upon individuals.

“Living well means striving to create a good life, but only subject to certain constraints essential to human dignity.” Whereas well-being is a static concept definable in terms of the set of minimal conditions that make up the good life “independently of the process through which it was created or of any other feature of its history.” In contrast to the notion of the good life, living well is a dynamic concept. How well one’s life goes is determined by the value of the “rising to the challenge of having a life to lead.” [18]

##### **Responsibility to the self**

It is objectively important to live well―compatible with the value of leading a critically good life whose estimate is not reducible to self-reported (i.e. subjective impression or experience of) well-being. There are objective standards on what a critically good life constitutes. In principle, a good life does not imply that one lives well and vice-versa. A person may be said to have summarily lived a good life with no or minimal striving to do so; and, on the other hand, one could be said to have lived well even though his was a life lived in poverty and misery. A Machiavellian prince who lived in material abundance and cultural and artistic sophistication may represent the first. Conversely, one could name a Van Gogh, Nietzsche, Nicola Tesla, or a William Blake as an epitome of the latter type.

However, it is appropriate to issue one caveat here: the unexamined life often undermines a critically good life. That is because one who leads a life without meaningful relationships, projects or challenges, without passions or a critical conception of the good that one deems worth pursuing—marking time to his or her death simply with an endless pursuit of hedonistic pleasure, has nevertheless not had a good life. Life is not supposed to just be good (defined in terms of one’s subjective impression of well-being), but critically good. This is therefore to say that, the ultimate value of life is adverbial; it is characterised by the performance value of striving for a good life, which is radically different from a life entirely devoted to prudential avoidance or minimising the chances of living a bad one. To live well one should venture out and engage in the adventures of life and risk having a bad one.

Living well presupposes an objective measure of the good life. On the flip side, disregarding one’s responsibilities to strive for a life of meaning is in turn detrimental to one’s actual well-being.

##### **Duties to others**

Analogous to the two distinct ways in which living well connects with well-being, our moral duties to others also connect with ethical responsibility in two distinct ways. The first is that, discharging one’s moral duties to others is one plausible path through which the person can live well. That is to say, pursuing a moral life is something individuals should have reason to value and strive to live accordingly. Secondly, moral life produces critical goodness to the life of the moral-agent herself.

One’s duties to others may relate either to the provision, protection and promotion of the essential components to their well-being, or to respecting the things in life that they have reason to value. The provision of basic health care through the system of UHC signifies the first, whereas respecting and fostering autonomy and dignity may signify the latter. In most cases our overriding moral duty relates to the second, namely to what makes their lives go well in contrast to the simple advancement of their well-being. The ethical primacy of living well over well-being warrants prioritising respect for dignity and autonomy over the advancement of wellbeing. We certainly have a responsibility to promote the well-being of others, but we have an overriding duty of respect for the things that render the lives of others go well even if that turns out to be detrimental to their wellbeing. Our moral duties as individuals sets the background for what we are collectively required to do via public policy.

We now proceed to showing how the above reflections can help unpack and critically analyse the conceptual model for *Healthy Ageing* adopted by the World Health Organization.

### Healthy ageing and the two distinctions in ethics and morality

The WHO framework for healthy ageing appears to reflect, albeit implicitly, the above described crucial distinctions in ethics in the manner commensurate with public health policy. In particular, *The World Report on Ageing and Health* defines healthy ageing as “the process of developing and maintaining the functional ability that enables well-being in older age.” Using two dynamic concepts, namely intrinsic capacity (IC) and functional ability (FA), *the Report* outlines a systematic approach for objectively improving the well-being of older persons that also takes seriously the significance of living an authentic life characterised simply as the sort of life older people have a reason to value. Accordingly, intrinsic capacity is defined as the composite of all the physical and mental capacities that an individual can draw on at any given point in his or her life. Functional ability, on the other hand, is characterised by a set of health-related attributes that enable people to be and do what they have reason to value; it is composed of the intrinsic capacity of the individual, relevant socio-environmental determinants and the interaction of the individual with those characteristics [19].

The next logical step is to ask: what, if anything, we owe to older persons with respect to their health and well-being? A generic answer may be that, what we owe to older persons must be consistent with the recognition that the ultimate value of life is adverbial, that is, the performance value or the ‘rising to the challenge of having a life to lead.’ It ought to reflect the value that older persons are not “simply passive vessels in which a good life may or may not occur”, and that “having a bad life does not always mean not having lived well.” [20]

Our concern for the health and well-being of older persons ought to be motivated by the same understanding of value such that the fundamental reason for improving their health and well-being is to foster the pursuit of meaning, as attested by a reasonable normative control, in their own life. Respect for older persons requires that we take seriously their right to pursue a life according to what they have reason to value, regardless of their station in life or their current health status. Our basic moral responsibility regarding their health should not therefore be constrained by their chances for leading an objectively good or bad life but must relate to their (functional) ability to live, a possibly good or bad life, well. That is to say that the moral significance of having a good life is ultimately grounded on the extent to which creating a good life contributes to living well, i.e. to the struggle to live according to what one has a reason to value.

* The phrase that one has “a reason to value” can carry two contrasting meanings: either in the descriptive (explanatory) or in the normative sense. In the descriptive sense, it may mean that the person is living a life which he actually values commensurate with a non-arbitrary value system. Having a reason to value can, on the other hand, be normative in the sense that it designates the life one values as one ought. In this sense, ‘having a reason’ implies a criterion for what counts as an objectively good life, or at least what is reasonable to value as such.

The capabilities approach advanced by Amartya Sen, adopts the first interpretation arguing that public policy should preserve and promote the capacities to function in ways consistent with negative freedoms, which means either abstaining from interfering with or removing obstacles from, the individuals’ free adoption of life paths and value systems that they deem for themselves as reasonable. This approach to value is concerned only with those capacities to perform valuable functioning that serve as the legitimate basis for government action and therefore warrant protection via public policy. One advantage of this approach is that it recognises that there is a social determinant to what individuals may actually have a reason to value.

Two points warrant making here. First, with respect to the WHO’s account of healthy ageing, we should interpret references to “having a reason to value” primarily in the descriptive sense of the term (connoting citizens’ minimally reasonable actual preferences). Secondly, the capabilities approach can accommodate the value of negative freedoms (“freedoms-from”) including respect for autonomy and dignity―those moral side constraints set up to safeguard the person’s normative control over central domains of one’s life. It is plausible to include these essential safeguards to the individual’s capability to function as appropriate policy goals, without having to concede that such freedoms-from are, themselves, capabilities. However, although the capabilities model confers a plausible guidance to public policy, it is however incomplete without an underpinning moral theory that establishes side-constraints as ends in themselves which will consequently set the criteria for determining when health inequalities are unfair or wrongful. Therein lies the significance of this paper.

A plausible account of the underpinning moral theory must begin with the recognition that one’s opportunity for leading a good life is partly influenced by the social, economic and political circumstances into which the person is born. And it goes without saying that, with respect to safeguards to personal freedom and a life worthy of human dignity, natural endowments and one’s initial place in the social strata (excluding hierarchies that are grounded in competence) are arbitrary from the moral point of view. In a decent society, regardless of their initial station in life, individuals will be granted the opportunity to create for themselves a life that is both minimally good and optimally functional. This moral dictum is also pertinent to arbitrary distinctions based on age.

Facts about the ageing process can also inform our moral point of view. Naturally intrinsic capacity (IC) tends to decline with increasing age; nonetheless, a mix of personal, environmental and structural factors will determine each individual’s trajectory of functional ability (FA). As it happens, for those with declining or significant lose in capacity due to multiple morbidities generally considered to be incidental to ageing, proper interventions at different points during their life course can improve and foster all-things-considered positive value in well-being sufficient for living well. Having a bad life due to declining health does not always imply not having lived well. That is to mean, it matters to have normative control over one’s life despite advanced age and even under the circumstances of severe decline in capacity.

Health is one crucial aspect of well-being, and as a basic human need and right, it certainly ought to be protected, promoted and advanced. Yet the significance of a long and healthy life is both inherent as well as instrumental, that is to say there is no point to prolong life for the sake of going on living unless we subscribe to the underlying notion that longer and healthy life opens doors for a life of meaning, vitality and excellence–that is a life worthy of human dignity, of which poor health or a life cut short could undermine or foreclose. It implies that, the responsibility of every society to improve the health and well-being of all its members is underpinned by the normative priority for what constitutes living well (a life worthy of human dignity). This certainly means that not all health concerns matter equally. Similarly, Daniel Hausman wrote that “how much on average a health deficiency matters to individuals need not equal the extent to which that deficiency matters to public health policy.” [21]

### Healthy ageing characterised as a life worthy of human dignity

There are two mutually reinforcing components to a life worthy of human dignity: authenticity and self-respect. Authenticity reflects a “special, personal responsibility for identifying what counts as success” in one’s life and the striving to lead such life “through a coherent narrative” commensurate with one’s own image of herself [22]. It requires that the person has autonomy over the essential domains of his or her life. Self-respect requires that one takes seriously the objective and intrinsic importance of one’s living well, which entails recognising the objective and equal importance of living-well for oneself as well as recognising the same for others. Thus, self-respect integrates our moral duties regarding others with one’s own ethical responsibility for living well. Certainly, self-respect permits that “I can respect others and hold that their lives is as objectively important as mine without at the same time taking equal interest and investment in their lives as I do in my own.” Such basic recognition for the objective significance of the lives of others can plausibly serve as a common thread that sets the moral background for public health policy. For instance, a health policy is deemed implausible if its provisions or omissions are predicated on the denial or infringement of respect to the objective significance of the lives of older people.

Self-respect and authenticity warrant the dignified respect and difference with which we ought to treat persons irrespective of the morally arbitrary features that drive a wedge between them. Moreover, the argument from dignity reflects that what we may be morally permitted, required or prohibited to do to others, and in particular to older adults, set the background for what we may be permitted, required or prohibited to do to them through the apparatus of the state [23].

Our duties regarding older persons, may either be general or specific, respectively reflecting both the respect they are owed as equal members of the moral community and the imperative to safeguard their specific vulnerabilities to various forms of indignities. That may involve (i) the duty of care, (ii) the duty not to create unreasonable risk of harm to older people including the impermissibility of deliberately causing them harm, (iii) the limited duty of forbearance from imposing unintended but foreseeable harm on them while pursuing other goals, and (iv) treating older persons in ways that denote a manifest denial of their equal moral status. In making policy decisions that concern the health of older persons we must keep in mind the difference in normative force between violating a negative duty (not to intentionally harm or wrong others, represented by ‘ii’ and ‘iv’), and infringing on our positive duties by mere omission [24].

However, infringement of a positive moral duty is not axiomatically less troubling than a violation of negative duties. Disinclining to discharge one’s duty of care for older persons, for instance, might engender an attitude towards them as if their life is of less moral worth than their younger fellows. This example illustrates that some refusals to discharge one’s duty of care are morally impermissible, if done for reasons that clearly signify the view that older persons count for less.

A case in point is the cost-effectiveness analysis that was specifically applied to renal dialysis in Thailand. For acute cases of kidney failure, the availability of dialysis often proves to have life-saving significance for the patient. But it certainly is far too expensive in resource-poor settings, costing 30 times the GDP per capita per healthy life year in Thailand. To put that in perspective, the cost for dialysis in Thailand is equivalent to 300 times as many healthy life years if spent on TB interventions, which commonly benefits younger age groups [25]. The principle of human dignity prohibits the refusal to accord older persons equal moral status which would be the case if Thailand were to prioritise investment on TB interventions based solely on the grounds of cost-effectiveness (presuming that kidney failure disproportionately affects older adults). What is more, the reasoning that underpins the specific cost-effectiveness metric (i.e. in terms of gain in healthy life year per capita) appears to conflict with the principle of respect for human dignity and its underlying moral ethos. That is to say, it may lead to policies that do not regard the lives of all citizens as holding equal moral standing.

When used as the principal criteria for selecting programs for a national health policy, cost-effectiveness estimates sometimes conflict with some basic moral principles, primarily with the principle of respect for the dignity of persons [26]. If we take, for example, end-of-life palliative care for older patients, it may appear as strikingly far too expensive than a society might be willing to invest particularly if the value of such care is defined in terms of gains in healthy life years in contrast to alternative (and perhaps more invasive) medical interventions. But if societies decide to divest from it in favour of services that could potentially earn more healthy life years either for older persons themselves or for others, such a decision will certainly amount to estimating the inherent value of persons on the basis of health outcomes potentially leaving older persons vulnerable to the kind of treatment as if they count for less or nothing at all. As a basic moral principle, respect for the dignity of persons generates that crucial litmus test for determining whether and when cost-effectiveness analysis is a permissible criterion for priority setting.

To put the that in context, in most sub-Saharan countries non-profit foundations constitute the major source of institutional care for older people (discounting integrated family care). That clearly indicates the relative neglect given to the care of older persons. Moreover, research has indicated that such model of care is unsustainable. The system relies primarily on volunteer care-givers, and services are resourced through cash and in-kind donations in the form of geriatric training, medical supplies and technical support. For instance, the Care for Aged Foundation in Ghana which operates mainly within Ga East municipality holds 3000 older people on a waiting list. Similarly, HelpAge International’s Better Health for Older People in Africa programme in the United Republic of Tanzania manages to support only 4500 older people all over the country, with care provided by 450 trained volunteers. Although Mauritius, Seychelles and South Africa have made great strides in investing on the health of older persons, the provision of care still falls short of demand while continued expansion will prove difficult to sustain given the general lack of commitment [27].

Similarly, respect for autonomy is another moral principle that figures prominently within the discourse on interpersonal moral duties which also seamlessly coalesces with the individual rights persons have against their political community. Earlier, autonomy was described as a normative condition for authenticity―the second essential component to a life worthy of human dignity. According to some promising accounts of personal autonomy, that there are two dimensions to an autonomous life: the *social dimension* of autonomy and the *temporal dimension*. The first proclaims that the social environment that one lives, and the personal relationships and deep attachments one establishes not only affect how one exercises her autonomy but that they are constitutive to it. And secondly, the identification of autonomous persons as self-sufficient rational choosers who can self-determine their own destiny must also take into account the fact that individuals exercise their autonomy over time [28].

#### Autonomy and healthy ageing

The autonomy of older persons should be conceptualised along the social and temporal dynamics. What this means in that, considering that autonomy is constitutively relational and is exercised over time (diachronic), a reduced intrinsic capacity and functional ability at older age does not necessarily imply a proportionally diminished value in autonomy. We can promote and enhance older persons’ autonomy by improving the environmental dynamic in which they live. Moreover, a plausible, and empirically informed, public health policy must take into account the fact that individuals (particularly true for older persons) exercise their autonomy over time.

Such understanding of autonomy can strengthen the WHO’s conceptual framework for healthy ageing and be of vital importance to developing person-centred and integrated care, including a nuanced policy on dementia and on the provision of palliative care. It does that by challenging the received view according to which social engagement is merely instrumental to enhancing autonomy. Misconceptions about the nature and value of autonomy reinforce ageist norms and practices either by overlooking older people’s need for social engagement as a basic constituent to their autonomy or by erroneously thinking that an episodic loss in the capacity for autonomy, for instance due to an onset of dementia, amounts to automatic termination of their right to autonomy.

However, the idea that autonomy is exercised over time should not be construed as an argument for ignoring older person’s current state of mind, desires and choices. One’s episodic choices ought to still be respected, including a range of choices from simple matters that pertain to organising one’s daily routine to matters of grave consequence such as choices relating to unbearably painful and invasive medical procedures. These legitimate interests in autonomy crucially inform the moral limits to paternalistic interventions in the name of older person’s well-being.

#### Human rights and healthy ageing

A human right can simply be defined as any fundamental right that we have in virtue of our basic equality as human beings, regardless of one’s accident of birth including membership to society. Human rights protect what is considered to be essential to a life worthy of human dignity. A human right-claim is not contingent, in the sense that it can be earned or granted and so in the same way can be forfeited or withdrawn. It is often declared that human rights are inalienable to the human person, that is to say, even if a person’s human right is impermissibly violated one does not thereby lose that right [29]. The moral grip that human rights have is, therefore, categorical. The right to health is only indirectly referred to under Art. Twenty-five of The Universal Declaration of Human Rights, which declares that “everyone has a right to a standard of living adequate for the health and well-being of himself and his family.”

The primacy of living well and the inalienability of human rights both reflect the normative separateness of individuals. We have seen earlier how the interpretation of living well yields a plausible route to the identification and grounding of our duties to others, and consequently hold that living well requires human dignity. In a strikingly similar way, UDHR is premised on the idea that human rights “derive from the inherent dignity of the human person.” However, a human right to health is too vague a concept that it permits multiple interpretations over its precise scope and normative grip, in which case the most reasonable approach is to investigate the extent to which health inequalities signify social and economic injustices. Opportunities to a healthy life is one of the areas in which societies can insure that substantive inequalities due to circumstances of birth, social and environmental factors do not morph into injustices; hence**,** the right to health and well-being of older persons can be approached from that general vantage point, precisely as one of the principal yardsticks of the just society.

If, on the contrary, we want the human rights idea to be distinctively informative about the claims peculiarly held by older persons, we ought to primarily identify the specific prohibitions and limitations prescribed by the rights approach to health and look to see if any of those moral prohibitions exclusively protect older adults [30]. References to ageing and older people have traditionally been limited within international human right treaties and instruments. But human rights language is increasingly being integrated into the topic of ageing and health. Notably, the Madrid International Plan of Action on Aging (2002) emphasises the imperative to safeguard for older persons the rights and freedoms enshrined under the international human rights instruments, including “the elimination of all kinds of violence and discrimination against older persons.” [31] In any case, a rights approach to healthy ageing must minimally recognise the following list of rights, some of which can be found listed under the UN Principles for Older Persons: right to a dignified life in older age; rights to liberty, independence and autonomy; rights to care and safety; the right to universal, affordable and quality health care; right to give free and informed consent on health matters; rights of older persons for and in receiving long-term care; right not to be subjected to cruel, inhuman or degrading treatment or punishment, while under institutional or home care; right to community participation; and equality and non-discrimination for reasons of age, including inclusion in health research and the right to participate in clinical trials.

#### Healthy ageing and social justice

At the outset of this section, we noted that there are two analytic routes towards conceptualising the nature, content and justification of the basic moral claims older persons have against others and society that are impermissible for trade-offs. In the preceding paragraphs, we attempted to shade light on the topic by adopting the second theoretical approach which required clarifying basic conceptual distinctions necessary for establishing a substantive moral theory according to which specific judgements about the inviolable claims of older persons are to be made.

In what follows, we quickly return to the first approach that seeks to identify unacceptable trade-offs attendant upon the health and well-being of older persons on the grounds of fairness. Health inequality is 1 area of concern for social justice, “it certainly is a very important part of our understanding of health equity, which is a broader notion.” [32] Inequality in health outcomes, although relevant, is not in itself indicative of injustice or inequity; it is therefore more promising to evaluate (the violation of) justice in health first by looking at whether institutions of society generally adhere to procedural fairness, i.e. the process through which health outcomes are brought about. For that reason, equality of opportunity in health care has inescapable relevance to social justice in regard to health. It is pertinent to note that health equity is a complex concept that includes concerns about health outcomes, the capability to achieve good health, procedural fairness in distribution of health care and the interplay between the analysis of inequalities in health and broader issues of social justice [33].

In contrast to the human rights approach to healthy ageing, social justice requires that one belongs to a political community and is owed recognition and difference in virtue of that membership. For human rights approach what is at stake is our human fellowship and our duties to each other in that regard, while social justice confers grounds for partiality to one’s own (nationals) compatriots in their capacity as free and equal members of a given society. Conceptualising health equity within the broader concerns of social justice provides a convenient platform for examining the pathways through which socio-economic and social determinants of health inequalities generate health inequities, and thus warrant remedial action [34]. It is important not to conflate principles that underpin global equality of opportunity for health at older age with considerations of social justice relevant to similar concerns of fairness in health within national boundaries. In general, global responsibilities to overcome unfair barriers to the health of older adults across nations tend to be weaker and less robust in contrast to the self-same responsibilities within a given nation; what is more, the distinction in normative force between the international and within national contexts may also generate a difference in focus with respect to specific areas of concern that warrant action.

John Rawls’ theory of *Justice as Fairness* provides invaluable insight in that regard. He defends two principles of justice, namely: *the principle of equal liberty*, and *the difference principle* underwritten by a fair *equality of opportunity* [35]. The second principle is more pertinent to the topic of specific concern to this paper, namely the proper moral demarcation between permissible and impermissible inequalities of concern and moral weight between the well-being of older persons and people in other age groups.

The principle of fair equality of opportunity justifies the importance of establishing a framework of universal health coverage. The difference principle, on the other hand, declares that relative inequalities in socio-economic status (the social determinants of health) are permissible only to the extent that they serve the least advantaged groups in society to be as well-off as possible. Consequently, the difference principle has a role to play in regulating the path to UHC. Inequalities in health status among individuals can be addressed simply by prioritising the well-being of the least well-off (it seems plausible to include older persons within the category of the least advantaged groups due to the overall decline in their powers of self-direction). The principle confers priority to the least advantaged subject to the proviso that such prioritisation does not undermine the value of equal liberty or the requirement of fair equality of opportunity (ex. UHC), reflecting the lexical ordering of principles [36].

Countries with an established national legal recourse to universal health coverage still need to address key issues of fairness attached to socio-economic determinants of health, which includes setting a benchmark for the provision of the social basis of self-respect without which individuals will have critically diminished overall functioning. There is on exhaustive list of what constitutes the social bases of self-respect, but any plausible theory of self-respect will inescapably have to make recourse to the concept of living well.

In practical terms, the ethical framework advanced here can underpin the moral salience of taking seriously the social reality in which many older persons in the world live. The distribution of the benefits and burdens of social life figures as the principal topic of social justice, and older-persons’ vulnerability must be weighed higher to potential positive benefits due to their respective implications for living well. Research has indicated that in most parts of the developing world vulnerability of older persons has been more severe than meets the eye, and even where sufficient data is available the problem has often times been overlooked by policy makers. The domains that shape vulnerability at older age must be analysed in ways that we can clarify pathways to “bad ends” and to identify possible points of intervention. In general, the framework of vulnerability in later life follows the following path: it begins with the risk of being exposed to a threat, where the probability of a threat actualizing into a harmful outcome depends on factors that either enhance or erode the person's defensive shields or coping mechanisms. Therefore, an older person's risk of harm may begin with exposure to threats but a bad outcome (in terms of reduced well being or health status) is a compound effect of such exposure and socio-economic determinants that shape an individual's capacity to cope with the threat to which one is exposed [37]. 

Each stage in the vulnerability function is constituted by structural and relational determinants. Structural vulnerability has to do with the socio-political and institutional infrastructures that may affect the capability of older persons to withstand difficulties; social realities of urban migration in majority agrarian societies, inadequate infrastructure including health care in rural areas, housing, poverty, and low quality of environment may count as domains of structural vulnerability. Whereas inadequate social networks or lack thereof, loneliness, discrimination and social marginalisation (on the basis of age, sex, disability, ethnicity and religion) may count as principally relational vulnerabilities. Both types of vulnerabilities often figure in tandem, as for example:

“83 per cent of Ethiopia’s population lives rurally, but migration to urban areas for work, family support and medical care increasingly brings older persons to city centres. Regardless of location, though, Ethiopia’s older persons are vulnerable to poverty, food insecurity, limited access to social and health services, and limited options for livelihoods diversification and security. They are further subject to the double protection bind of both needing care and protection in their older years and needing to support children, grandchildren, and ageing spouses in their care. The impact of the HIV pandemic combined with acute economic stress has resulted in changed family structures across Ethiopia. The loss of middle generations has created family structures where almost half of Ethiopia’s orphaned children are cared for by grandparents.” [38]

There is a growing realization that structural and relational factors disproportionately affect older persons partly due to their reduced functioning and partly because of the relatively low priority they receive in developmental and social safety net programs. The above example illustrates not only the significance of social realities in tailoring the contextual application of principles of social justice but also the ethical implications of the failure of society to address them. What that means is that success or failure can drive a wedge between just and unjust societies. With respect to decisions about public policy in resource scarce contexts where a slight alteration would have life altering consequences, decision makers must therefore take seriously the lexical ordering of principles of social justice and confer priority to the least well-off members of society.

## Impermissible trade-offs and the ethical priority for living-well

This paper recognises the validity of the five unacceptable trade-offs identified in the final report of the WHO Consultative Group on Equity and Universal Health Coverage [39]. It also recognises that those five unacceptable trade-offs are established on broader grounds of justice as fairness and the special concern it confers to the least advantaged. The ethical framework defended here can underpin some of them, whereas the rest are stipulated in terms of general principles of practical reason and can therefore be established without the need to make explicit recourse to moral considerations.

We shall not pursue a pointed remark on each of the five unacceptable trade-offs, partly for reasons of space and partly for the reason that they are not designed in the first instance to address our specific concern for the health and well-being of older persons [40].

The ethical priority for living well, and the central role that respect for human dignity plays in articulating as well as underpinning our moral duties regarding others, sanctions against discriminating persons from the scheme of UHC on arbitrary grounds such as their designated place in the social hierarchy. It matters from the moral point of view that we take seriously the inherent worth of all human beings, regardless of their station in life.

The concept of living well has both general and specific interpretations. Generally living well consists in striving to make a life that one a reason to value. But in specific terms, what constitutes living well for young adults differs in in content from what living well might consist in for older persons. Early adulthood epitomises the point at which one’s capacities for self-direction begins to peak, whereas a declining trajectory in capacities is typically correlated with older age. The inherent quality of their respective life, therefore, necessitates a specific articulation of what it means for each category of persons to live-well. “Young adult” and “older person” are phase sortals, in the same way “caterpillar” and “butterfly” are. When we reach the age of 65, we ceased to be adults *simpliciter*, but we don’t thereby cease to be persons. Such specification does not, however, contradict whatever normative work living well does in general–underpinning the categorical and non-optional ethical responsibilities to oneself, in addition to grounding the moral primacy of respecting human dignity and autonomy over the provision of basic needs.

Human dignity, as does the idea of living well, manifests a general-specific Janus face. As a general normative concept, human dignity protects persons from humiliating and degrading treatments including the prohibition of treating persons merely as a means or as if they morally count for less or nothing at all. It does these works for older people as well. But the exact content of these normative functions ought to be articulated in accordance with what it means for older persons to live well and lead a life of dignity and authority.

The inherent quality of older age can be captured by two distinctive features: the first relates to the familiar association of ageing with non-trivial decline in capacities, and the second is the fact that at older age attributions of value and meaning to one’s life must ultimately take the point of view of the entire spectrum of life–including not only the life lived up to the point older age eventuates but also a reasonable account of what is in store for him/her until life’s eventual ending. In relation to that, a plausible conception of human dignity ought to elucidate the normative significance of the capacities that survive the general decline attendant upon ageing, determining the threshold for decline in the capacity for self-direction that would leave the dignity of older persons intact. Similarly, we ought to evaluate the significance of preserving dignity at older age not only for their pursuit of meaning at that particular juncture in their life but at the same time for the preservation of value and meaning of life as a whole.

### Three impermissible trade-offs

Viewed from both the general and specific modes of construal, we submit that the ethical primacy for living well underpins that the following trade-offs relating to the health and well-being of older persons are morally impermissible.

1. It is impermissible to prioritise services across different age groups on the basis of actual or future contribution to society
  This needs some unpacking. If, for instance, two comparable sets of health services or interventions need prioritising and that each presumably target different age groups with relatively comparable scale of vulnerability, it will be wrongful to employ however implicitly the *social worth* criteria according to which actual or potential future contribution to society dictate the principal focus of social investment. The ethical framework defended in this article rejects as impermissible the utilisation of the *social worth* criterion in the determination of health care priorities across age groups.
  The equal objective moral significance of each individual’s life must not be forsaken on account of differences in social worth amongst groups of individuals. Evidently, some segments of society such as women and children, and people on the lowest economic strata are more vulnerable than others. Such differences on the scale of vulnerability can be an adequate criterion for selecting services into priority classes but cannot be utilised for prioritising services across age groups. If we were to choose between two life-saving interventions, one benefiting infants, children or young adults and the other older persons, it appears to be intuitively plausible to favour the younger age group over older persons. Such preference has been the modus operandi of health policy financing both at the national and international levels. But the intuitive drive to put women and children first in health care policy suffers from an implicit bias towards perceptions of social worth. Concrete examples can be found in many national health service strategies. For instance, Ethiopia’s essential health service package (ESHP) aims to provide essential health care primarily targeting free coverage for tuberculosis; maternal care and family planning; immunization services; HIV/AIDS; leprosy; fistula; and epidemics (Federal Ministry of Health 2005). The omission of non-communicable chronic diseases, typically affecting older persons, demonstratively reveals implicit biases in favour of younger age groups. When done at the expense of others, such pattern of prioritisation constitutes a violation of the inherent dignity of the human person. Certainly, sustainable investment in maternal, new born or child health programs are noble pursuits; but the point highlighted here is that, that should not be done at the expense of older adults who in some measure also count amongst the most vulnerable age groups.
2. It is impermissible to prioritise across age groups when co-prioritisation is warranted by the ethical theory
  This rule is a logical consequent of the point illustrated by the above example. The process of defining high priority services is generally neutral about age group, gender, health status, and other markers of distinction generally considered to be arbitrary from the moral point of view. One example maybe the provision of anti-HIV drugs. In terms of healthy life years, these drugs greatly benefit younger patients as opposed to the benefit in healthy life years for older adults. People accrue the full benefit of these drugs if they are younger and are expected to have more healthy life years given the current life expectancy. However, it is unacceptable to prioritise coverage for this service on the basis of age. A plausible health policy that takes seriously the equal inherent worth of all human beings must by definition co-prioritise life-saving and life sustaining drugs or interventions regardless of the amount of the actual gain in healthy life years for the individual. On the horizontal dimension of co-prioritisation, one can speak about services that should be conferred equal status within a given age group. Evidently, both infectious and non-communicable diseases affect older persons and can equally be life threatening. In this case, co-prioritisation is warranted by the moral theory. However, the Ethiopian essential health services package (a national baseline for UHC) has ignored the latter in favour of infectious diseases. Perhaps the underlying reason has to do with the fact that infectious and communicable diseases have crosscutting effects, hence requiring precedence from the public health point of view. Whereas, the moral framework defended in this paper overrules public health norms and practices when they conflict with the equal worth principle.
3. Services that improve well-being ought not always get primacy (by default) over services that foster autonomy and dignity. Contrariwise, the general rule is that respect for autonomy and dignity hold primacy over services that improve well-being at the expense of the first.
  It is impermissible to prioritise critically important services that may add more life years to older adults if the added years in functioning imply a life spent with indignity, severely degraded autonomy and normative control over the essential domains of one’s life. Here’s where the conception of the dignity of older persons adopts the specific construal. At older age, an improvement in health and well-being must follow the path of dignified daily functioning. That is to mean, regardless of overall benefits in improving well-being any health intervention that severely compromises older persons’ psychological and bodily integrity is considered wrongful, hence impermissible, from the moral point of view. For instance, in line with many customary sub-Saharan norms, family solidarity and obligation constitute the core elements of older people’s understanding of their own autonomy and dignity [41]. Inter-generational relations and perceptions of one’s legacy are considered to be key to older persons’ self-respect. These norms favour strengthening long-term home care for older persons, since it perceptively preserves older persons’ sense of dignity and autonomy. However, research also indicated that institutional care is more conducive for preserving and improving health related well-being, while older persons at home care are more susceptible to elder abuse, even in African societies, than meets the eye [42]. In this scenario, the ethical framework defended here favours prioritizing long-term home care despite its reduced effect on preserving wellbeing in comparison to institutional care. One caveat is that the framework for long-term home care should include innovative safeguards against elder abuse.
  There is one caveat here, which is that, in that context a second-best choice must be available which can balance an improved well-being with the maintenance of dignity and autonomy. Even the seemingly positive idea of quantifying the value of a health policy option in terms of the added “healthy life years” that it makes available to the individual can have catastrophic consequences if adopted for priority setting. Suppose for example, there is a certain intervention X for a terminal medical condition Y that may be known to be effective in adding 12 relatively healthy life years to an older person but at the cost of a loss in just one functioning that renders older persons incontinent throughout the entire added life years. Now suppose that there is an alternative costly intervention Z that adds a comparably meagre 4 healthy life years but without the indicated side effect. Although on average well-being is greatly improved under X, however, considering the possibility of leading an autonomous and dignified life, it is impermissible to deny older persons the opportunity to opt for Z.

## Conclusion

The claims defended here in this paper pivot on one underlying thesis, which is that the public provision of health ought to reflect the idea that older persons have an equal moral standing. This paper prescribes that we take seriously that preserving older persons’ specific dignity partly requires retrospective look back at their past such that we are required to treat them in ways that do not degrade their enduring status as rational and autonomous members of the moral community. Although the general framework of dignity and autonomy remain the same, what makes an action or behaviour autonomous differs in content depending on to whom it applies. A simplistic set of markers may adequately explain what counts for a pre-adolescent child to be autonomous (given that in most countries the legal age of maturity is traditionally set for the age of 18). Whereas in adulthood, a more stringent criteria maybe made applicable. However, a person suffering from dementia will not suddenly lose his rights for autonomy. Instead, the onset of dementia compels the requirement for a reflective adjustment in the cognitive, behavioural and volitional markers of autonomy. This is therefore to say that, we should treat older persons with recognition and difference to the fact that progressive decline in capacity and functioning does not imply a loss or decline in their moral worth on which their fundamental rights and dignity are predicated.

## Data Availability

Not applicable.
